# Health-Risk Behaviors and Chronic Conditions Among Adults with Inflammatory Bowel Disease — United States, 2015 and 2016

**DOI:** 10.15585/mmwr.mm6706a4

**Published:** 2018-02-16

**Authors:** Fang Xu, James M. Dahlhamer, Emily P. Zammitti, Anne G. Wheaton, Janet B. Croft

**Affiliations:** ^1^Division of Population Health, National Center for Chronic Disease Prevention and Health Promotion, CDC; ^2^Division of Health Interview Statistics, National Center for Health Statistics, CDC.

Inflammatory bowel disease (IBD), which includes Crohn’s disease and ulcerative colitis, involves chronic inflammation of the gastrointestinal tract. In 2015, an estimated 3.1 million adults in the United States had ever received a diagnosis of IBD (*1*). Nationally representative samples of adults with IBD have been unavailable or too small to assess relationships between IBD and other chronic conditions and health-risk behaviors (*2*). To assess the prevalence of health-risk behaviors and chronic conditions among adults with and without IBD, CDC aggregated survey data from the 2015 and 2016 National Health Interview Survey (NHIS). An estimated 3.1 million (unadjusted lifetime prevalence = 1.3%) U.S. adults had ever received a diagnosis of IBD. Adults with IBD had a significantly lower prevalence of having never smoked cigarettes than did adults without the disease (55.9% versus 63.5%). Adults with IBD had significantly higher prevalences than did those without the disease in the following categories: having smoked and quit (26.0% versus 21.0%; having met neither aerobic nor muscle-strengthening activity guidelines (50.4% versus 45.2%); reporting <7 hours of sleep, on average, during a 24-hour period (38.2% versus 32.2%); and having serious psychological distress (7.4% versus 3.4%). In addition, nearly all of the chronic conditions evaluated were more common among adults with IBD than among adults without IBD. Understanding the health-risk behaviors and prevalence of certain chronic conditions among adults with IBD could inform clinical practice and lead to better disease management.

The NHIS is a cross-sectional household health survey of the civilian noninstitutionalized population. The survey provides nationally representative data on a broad range of topics, including health status, health behaviors, and access to and use of health care.* Data on diagnosed IBD (hereafter referred to as IBD) were collected with the Sample Adult Core questionnaire using the following question: “Have you ever been told by a doctor or other health professional that you had Crohn’s disease or ulcerative colitis?” The sample adult is randomly selected from all adults aged ≥18 years in the family and answers for himself/herself (unless physically or mentally unable to do so, in which case a knowledgeable adult serves as a proxy respondent). Interviews are conducted in respondents’ homes, although follow-ups by telephone to complete missing sections are permitted. To ensure more precise estimates of IBD status, the 2015 and 2016 Sample Adult data files were combined with the 2-year response rate of 54.7%.^†^

The prevalence of IBD, with 95% confidence intervals, was estimated for the civilian, noninstitutionalized U.S. adult population overall and by various sociodemographic characteristics. These characteristics, collected with the Household Composition and Family Core questionnaires, included age, sex, race/ethnicity, education level, marital status, current employment status, nativity, health insurance coverage type (reported separately for adults aged <65 and ≥65 years), urbanicity, and region of residence. Next, the prevalence of five health-risk behaviors^§^ (cigarette smoking status, binge drinking, body mass index [BMI] category, meeting of federal physical activity guidelines, and short sleep duration), serious psychological distress^¶^ (a proxy for mental health symptoms), and several chronic conditions** (cardiovascular disease, respiratory disease, cancer, diabetes, arthritis, weak or failing kidneys, any liver condition, and ulcer) were estimated separately for adults with and without IBD. All prevalence estimates met the reliability standard of relative standard errors <30%^††^ and were age-adjusted to the projected 2000 U.S. population^§§^ (unless otherwise noted). For comparison of IBD prevalence by subgroup and prevalence of health-risk behaviors and chronic conditions by IBD status, differences were considered significant if two-tailed Z-tests yielded p-values <0.05. All comparisons described in the results were statistically significant. All analyses were conducted using statistical software to account for the stratified, complex cluster sampling design of the survey. Estimates incorporated the final sample adult weights adjusted for nonresponse and calibrated to population control totals to generalize the estimates to the civilian noninstitutionalized population aged ≥18 years.

In 2015 and 2016, 3.1 million (unadjusted lifetime prevalence of 1.3%; age-adjusted lifetime prevalence of 1.2%) U.S. adults had ever received a diagnosis of IBD (Table 1). The age-specific prevalence of IBD was higher among adults aged 45–64 and ≥65 years (both 1.7%) than among those aged 18–24 (0.5%) or 25–44 (1.0%) years. The prevalence of IBD was higher among women (1.5%) than among men (1.0%); among non-Hispanic white adults (1.4%) than among non-Hispanic black adults (0.6%) or other non-Hispanic adults (0.8%); among those with less than a high school education (1.6%) than among those with at least a bachelor’s degree (1.1%); among those who were divorced, separated, or widowed (2.3%) than among persons who were married or cohabitating (1.1%); among currently unemployed (1.6%) or U.S.-born (1.3%) adults than their employed (1.1%) and non–U.S.-born (0.8%) counterparts; and among adults living in small metropolitan statistical areas (MSAs) (1.4%) than among those living in large MSAs (1.1%). The prevalence of IBD did not differ significantly among groups defined by health insurance coverage type or region of residence.

**TABLE 1:** Prevalence of inflammatory bowel disease* among U.S. adults aged ≥18 years, by sociodemographic characteristics — National Health Interview Survey, 2015–2016

Characteristic	Estimated no.^†^	Age-adjusted^§^ % (95% CI)
**Total (unadjusted)**	**3,121,000**	**1.3 (1.2–1.4)**
**Total (age-adjusted)**	**3,121,000**	**1.2 (1.1–1.3)**
**Age group (yrs)**		
18–24	152,000	0.5 (0.3–0.8)
25–44	798,000	1.0 (0.8–1.1)
45–64	1,394,000	1.7 (1.5–1.9)
≥65	777,000	1.7 (1.4–1.9)
**Sex**		
Men	1,219,000	1.0 (0.9–1.2)
Women	1,902,000	1.5 (1.3–1.6)
**Race/Ethnicity**		
Non-Hispanic white	2,363,000	1.4 (1.3–1.6)
Non-Hispanic black	174,000	0.6 (0.4–0.8)
Hispanic	427,000	1.2 (0.9–1.6)
Non-Hispanic other^¶^	157,000	0.8 (0.6–1.2)
**Education level**		
Less than high school	491,000	1.6 (1.2–2.0)
High school diploma/GED	748,000	1.2 (1.0–1.4)
Some college	971,000	1.3 (1.1–1.5)
Bachelor’s degree or higher	906,000	1.1 (1.0–1.3)
**Current marital status**		
Married/Cohabitating	1,823,000	1.1 (1.0–1.3)
Never married	484,000	1.3 (1.0–1.6)
Divorced/Separated/Widowed	814,000	2.3 (1.4–3.7)
**Current employment**		
Yes	1,538,000	1.1 (1.0–1.3)
No	1,583,000	1.6 (1.4–1.8)
U.S.-born**		
Yes	2,741,000	1.3 (1.2–1.4)
No	381,000	0.8 (0.6–1.1)
**Health insurance coverage** ^††^		
**Age <65 years**		
Private	1,578,000	1.1 (1.0–1.3)
Medicaid and other public coverage	354,000	1.4 (1.1–1.8)
Other	179,000	1.3 (0.9–1.7)
Uninsured	231,000	1.0 (0.8–1.4)
**Age ≥65 years**		
Private	338,000	1.7 (1.4–2.2)
Medicare and/or Medicaid	64,000	2.0 (1.2–3.1)
Medicare Advantage	215,000	1.8 (1.4–2.5)
Medicare only, excluding Medicare Advantage	104,000	1.3 (0.8–2.0)
Other	55,000	1.4 (0.9–2.4)
Uninsured^§§^	NA	NA
**Urbanicity** ^¶¶^		
Large MSA	1,542,000	1.1 (1.0–1.3)
Small MSA	1,366,000	1.4 (1.2–1.6)
Not in MSA	213,000	1.4 (1.0–1.8)
Region***		
Northeast	591,000	1.3 (1.1–1.6)
Midwest	752,000	1.3 (1.1–1.6)
South	1,092,000	1.2 (1.0–1.4)
West	686,000	1.2 (1.0–1.4)

**Abbreviations:** CI = confidence interval; GED = General Educational Development certificate; MSA = metropolitan statistical area; NA = not applicable.

* Respondents who had ever been told by a doctor or other health professional that they had Crohn's disease or ulcerative colitis.

^†^ The estimated annual numbers, rounded to 1,000s, were calculated based on 2015 and 2016 data. Counts for adults of unknown status (responses coded as “refused,” “don’t know,” or “not ascertained”) with respect to inflammatory bowel disease status are not shown separately in the table, nor are they included in the calculation of percentages (as part of either denominator or the numerator), to provide a more straightforward presentation of the data. In addition, frequencies presented in the table might be underestimated because of item nonresponse and unknowns.

^§^ Estimates (except for age groups and crude total) are age-adjusted using the projected 2000 U.S. population distribution #8 as the standard population and four age groups: 18–24, 25–44, 45–64, and ≥65 years. https://www.cdc.gov/nchs/data/statnt/statnt20.pdf.

^¶^ Non-Hispanic other includes non-Hispanic American Indian and Alaska Native only, non-Hispanic Asian only, non-Hispanic Native Hawaiian and Pacific Islander only, and non-Hispanic multiple race.

** U.S.-born includes all persons born in the United States or a United States territory.

^††^ Based on a hierarchy of mutually exclusive categories. Adults with more than one type of health insurance were assigned to the first category in the hierarchy. “Uninsured” includes adults who had no coverage as well as those who had only Indian Health Service coverage or had only a private plan that paid for one type of service such as accidents or dental care.

^§§^ In the survey sample, zero adults aged ≥65 years and uninsured had ever been told by a doctor or other health professional that they had Crohn's disease or ulcerative colitis.

^¶¶^ Large MSAs have a population size of ≥1 million; small MSAs have a population size of <1 million. Persons “Not in MSA” do not live in a metropolitan statistical area.

*** *Northeast*: Connecticut, Maine, Massachusetts, New Hampshire, New Jersey, New York, Pennsylvania, Rhode Island, and Vermont. *Midwest*: Illinois, Indiana, Iowa, Kansas, Michigan, Minnesota, Missouri, Nebraska, North Dakota, Ohio, South Dakota, and Wisconsin. *South*: Alabama, Arkansas, Delaware, District of Columbia, Florida, Georgia, Kentucky, Louisiana, Maryland, Mississippi, North Carolina, Oklahoma, South Carolina, Tennessee, Texas, Virginia, West Virginia. *West*: Alaska, Arizona, California, Colorado, Hawaii, Idaho, Montana, Nevada, New Mexico, Oregon, Utah, Washington, and Wyoming.

Being a former smoker was more prevalent among adults with IBD (26.0%) than among adults without IBD (21.0%), and having never smoked was less prevalent among adults with IBD (55.9%) than among those without IBD (63.5%) (Table 2). In addition, adults with IBD had higher prevalences than those without IBD of sleeping <7 hours per day (38.2% versus 32.2%) and meeting neither aerobic nor muscle-strengthening physical activity guidelines (50.4% versus 45.2%). No statistically significant difference was detected in the prevalence of binge drinking or BMI category between the two groups. The prevalence of experiencing serious psychological distress was reported twice as frequently by adults with IBD (7.4%) than by those without IBD (3.4%). Among the selected chronic conditions, with the exception of diabetes, all were significantly more prevalent among adults with IBD than among those without IBD (Table 2). The prevalence of ulcer was nearly five times higher among adults with IBD (26.0%) than among those without IBD (5.5%).

**TABLE 2:** Age-adjusted prevalence of selected health-risk behaviors and chronic conditions by inflammatory bowel disease* status among U.S. adults aged ≥18 years — National Health Interview Survey, 2015–2016

Characteristic	Adults with IBD		Adults without IBD	
	Estimated no.^†^	Age-adjusted^§^ % (95% CI)	Estimated no.	Age-adjusted^§^ % (95% CI)
**Cigarette smoking status** ^¶^				
Current smoker	557,000	18.0 (14.9–21.7)	36,561,000	15.5 (15.0–15.9)
Former smoker	949,000	26.0 (22.2–30.2)**	52,541,000	21.0 (20.6–21.5)
Never smoker	1,608,000	55.9 (51.3–60.5)**	150,357,000	63.5 (63.0–64.0)
**Drinking status^††^**				
Binge drinking (≥12 days) in the past year	250,000	9.8 (6.9–13.6)	22,207,000	9.9 (9.5–10.2)
**BMI groups (kg/m^2^)^§§^**				
Underweight (<18.5)	71,000	2.4 (1.4–4.0)	4,286,000	1.9 (1.7–2.0)
Normal (≥18.5 and <25.0)	1,007,000	35.9 (31.1–41.0)	78,296,000	34.2 (33.7–34.8)
Overweight (≥25.0 and <30.0)	995,000	31.0 (26.9–35.5)	79,812,000	34.2 (33.7–34.7)
Obese (≥30)	954,000	30.7 (26.2–35.6)	69,410,000	29.7 (29.2–30.3)
**Met physical activity guidelines** ^¶¶^				
Neither aerobic nor muscle-strengthening activity	1,680,000	50.4 (45.6–55.2)**	108,231,000	45.2 (44.6–45.8)
Aerobic activity only	770,000	25.4 (21.7–29.5)	68,340,000	29.2 (28.7–29.7)
Muscle-strengthening activity only	116,000	3.4 (2.0–5.5)	8,360,000	3.5 (3.3–3.7)
Both aerobic and muscle-strengthening activities	509,000	20.9 (16.9–25.5)	50,666,000	22.1 (21.7–22.6)
Less than 7 hours of sleep, on average***	1,138,000	38.2 (33.4–43.3)**	74,316,000	32.2 (31.6–32.7)
**Serious psychological distress** ^†††^	259,000	7.4 (5.4–10.0)**	8,161,000	3.4 (3.2–3.6)
**Chronic conditions** ^§§§^				
Cardiovascular disease	748,000	19.2 (16.3–22.5)**	31,229,000	12.0 (11.7–12.4)
Respiratory disease	870,000	27.3 (23.3–31.7)**	40,284,000	16.6 (16.2–17.0)
Cancer	547,000	13.7 (10.9–17.0)**	21,430,000	8.1 (7.9–8.3)
Diabetes	448,000	10.1 (8.2–12.4)	22,647,000	8.6 (8.4–8.9)
Arthritis	1,415,000	36.3 (32.8–40.0)**	55,114,000	21.1 (20.8–21.5)
Weak or failing kidneys	171,000	4.5 (3.2–6.3)**	4,703,000	1.8 (1.7–1.9)
Any liver condition	192,000	5.2 (3.7–7.2)**	4,207,000	1.7 (1.6–1.8)
Ulcer	800,000	26.0 (22.2–30.3)**	13,888,000	5.5 (5.3–5.7)

**Abbreviations:** BMI = body mass index; CI = confidence interval; IBD = inflammatory bowel disease.

* Respondents who had ever been told by a doctor or other health professional that they had Crohn's disease or ulcerative colitis.

^†^ The estimated annual numbers, rounded to 1,000s, were calculated based on the 2015 and 2016 data. Counts for adults of unknown status (responses coded as “refused,” “don’t know,” or “not ascertained”) with respect to IBD status are not shown separately in the table, nor are they included in the calculation of percentages (as part of either denominator or the numerator), to provide a more straightforward presentation of the data. In addition, frequencies presented in the table might be underestimated because of item nonresponse and unknowns.

^§^ Estimates are age-adjusted using the projected 2000 U.S. population as the standard population and four age groups: 18–24, 25–44, 45–64, and ≥65 years.

^¶^ Cigarette smoking status was defined as current, former, or never smoker. Current smokers reported having smoked ≥100 cigarettes in their lifetime and currently smoking cigarettes some days or every day. Former smokers reported having smoked ≥100 cigarettes in their lifetime but were not current smokers at the time of the survey. Never smokers reported they had not smoked ≥100 cigarettes in their lifetime.

** Statistically significant (p<0.05) difference between adults with IBD and adults without IBD.

^††^ Binge drinking ≥12 days in the past year was defined according to a response of the number of days to the question “In the past year, on how many days did you have 5 or more [for men]/4 or more drinks [for women] of any alcoholic beverage?”

^§§^ BMI was calculated as weight (kg)/height (m^2^) based on responses to the questions “How tall are you without shoes?” and “How much do you weigh without shoes?” BMI (kg/m^2^) was categorized as underweight (<18.5), normal weight (≥18.5 and <25.0), overweight (≥25.0 and <30.0), or obese (≥30.0).

^¶¶^ The definition of physical activity categories followed *2008 Physical Activity Guidelines for Americans* (https://health.gov/paguidelines/pdf/paguide.pdf). Both aerobic and muscle-strengthening guidelines are met if participants reported ≥150 minutes of moderate or ≥75 minutes of vigorous equivalent aerobic activity per week and muscle strengthening activities on ≥2 days per week.

*** Short sleep duration was defined as <7 hours in response to the question “On average, how many hours of sleep do you get in a 24-hour period?”

^†††^ Serious psychological distress is based on responses to six questions that ask how often a respondent experienced certain symptoms (feeling so sad nothing could cheer you up; nervous; restless or fidgety; hopeless; that everything was an effort; worthless) of psychological distress during the past 30 days. The response codes (0–4) of the six items for each person are summed to yield a scale with a 0–24 range. A value of ≥13 for this scale is used here to define serious psychological distress.

^§§§^ Cardiovascular disease included a history of any of the following conditions: coronary heart disease, angina, myocardial infarction, stroke, or any heart disease. Respiratory disease included a history of any of the following conditions: emphysema, chronic bronchitis, chronic obstructive pulmonary disease, or asthma. Cancer included cancer or a malignancy of any kind. Diabetes was defined as an affirmative response to the question “Other than during pregnancy, have you ever been told by a doctor or other health professional that you have diabetes or sugar diabetes?” Arthritis was defined as an affirmative response to the question “Have you ever been told by a doctor or other health professional that you have some form of arthritis, rheumatoid arthritis, gout, lupus, or fibromyalgia?” Weak or failing kidneys was defined as an affirmative response to the question “During the past 12 months, have you been told by a doctor or other health professional that you had weak or failing kidneys? Do not include kidney stones, bladder infections or incontinence.” Any liver condition was defined as an affirmative response to the question “During the past 12 months, have you been told by a doctor or other health professional that you had any kind of liver condition?” Ulcer was defined as an affirmative response to the question “Have you ever been told by a doctor or other health professional that you had an ulcer?”

## Discussion

Based on a nationally representative sample, during 2015–2016, an estimated 3.1 million U.S. adults had ever received a diagnosis of IBD. IBD might require lifelong disease management, including a combination of prescription medications, surgery, and medical treatment in outpatient, inpatient, emergency department, or ambulatory care settings. The symptoms and complications of IBD are associated with substantially impaired health-related quality of life (*3*). The total direct and indirect costs from loss of earnings or productivity attributable to IBD in the United States were estimated in 2014 to be $14.6 billion–$31.6 billion^¶¶^; however, because this estimate was based on a lower prevalence of IBD than that presented in this report, and given the impact of inflation, the current costs might be substantially higher.

In this study, the prevalence of IBD was higher among women, non-Hispanic whites, and older, less educated, and unemployed adults, which is consistent with the findings of previous studies (*1*,*4*,*5*). For example, in a previous study using insurance claims data, the prevalence of Crohn’s disease and ulcerative colitis was higher among older adults, and although the prevalence of ulcerative colitis did not differ significantly by sex, women were more likely than men to have Crohn’s disease (*4*). In this study, however, the survey question did not differentiate Crohn’s disease from ulcerative colitis. This study also found IBD to be more prevalent among unemployed adults, reinforcing previous findings on the employment burden of the disease (*5*). However, unlike other studies (*4*,*6*), no evidence was found of a difference in IBD prevalence by region of residence, which might be a result of different data collection modes and target populations in different studies.

Adults with IBD were more frequently former smokers and less frequently never smokers than were those without IBD. Some smokers might possibly have quit smoking because of a diagnosis of IBD. The role of smoking in the development of IBD is not fully understood. Smoking among persons with Crohn’s disease, however, has been found to be associated with disease development, progression, and inferior treatment outcomes (*7*). Smoking cessation, therefore, is particularly recommended among patients with diagnosed Crohn’s disease (*7*). Many chronic conditions are more common among adults who report a short sleep duration.*** Similarly, this study found that short sleep duration was more prevalent among adults with IBD. In addition, the prevalence of meeting neither aerobic nor muscle-strengthening physical activity guidelines was higher among adults with IBD, which might be an indication of severity of symptoms. Although there is no current exercise recommendation to adults with IBD, mild exercise in those with mild or moderate symptoms might not worsen disease symptoms (*8*). Furthermore, exercise might help build muscle mass, bone density, and improve sleep quality, and its benefits outweigh the risks for almost everyone. Adults with IBD who have mild to moderate disease activity should be encouraged to consult their clinicians about their exercise engagement.

Several chronic conditions were more prevalent among adults with IBD than among those without IBD. Although few comprehensive studies of IBD comorbidities exist, the disease has been found to be associated with multiple diseases, only some of which were gastrointestinal-related (*9*). For example, adults with IBD are at increased risk for certain cancers and osteoporosis (*7*). In this study, the prevalence of having experienced serious psychological distress in the last 30 days was higher among adults with IBD. This is consistent with past research that found adults with IBD have an increased prevalence of psychological or psychosocial disorders, including depression, anxiety, and impaired social interactions (*10*). Psychological disorders were also predictive of poor health-related quality of life, regardless of the severity of IBD (*10*). The presence of certain chronic conditions in addition to IBD might impair health-related quality of life among affected persons and further complicate disease progression and care management (*9*).

The findings in this study are subject to at least six limitations. First, because NHIS responses are self-reported and not corroborated by medical records, they are subject to reporting bias. Second, diagnosis of Crohn’s disease and ulcerative colitis could not be assessed separately as they are combined in a single survey question of IBD. Third, questions on other chronic conditions likely to be associated with IBD, such as anemia and osteoporosis, are not asked in the NHIS. Fourth, a short-term measure of serious psychological distress (within the last 30 days) was used as a proxy measure for mental health symptoms; therefore, the prevalence of serious psychological distress among adults with IBD could be underestimated. Fifth, although the sample weights include adjustments for survey nonresponse, the potential for nonresponse bias in the IBD estimates remains, given the Sample Adult Core response rate of 54.7% for the 2 years under analysis. Finally, the NHIS survey excluded active duty military personnel and institutionalized adults; therefore, the results cannot be generalized to the entire U.S. adult population.

Understanding the extent to which adults with IBD experience comorbidities helps further elucidate the impact of IBD. Further, assessing the health-risk behaviors of persons with IBD might aid in identifying opportunities to improve their overall health, quality of life, and disease management. Given the disease’s complexity and the effects of chronic conditions and symptoms, optimal IBD care might require a multidisciplinary approach that includes gastroenterologists, preventive medicine specialists, and other medical practitioners.

**Summary**

**What is already known about this topic?**

In 2015, an estimated 3 million U.S. adults had inflammatory bowel disease (IBD). The prevalence of IBD was higher among adults who were aged ≥45 years, white, U.S.-born, unemployed, and who had less than a high school education.

**What is added by this report?**

Based on 2015 and 2016 National Health Interview Survey data, being a former smoker was more prevalent and having never smoked was less prevalent among adults with IBD than among adults without IBD. In addition, meeting neither aerobic nor muscle-strengthening physical activity guidelines, sleeping <7 hours, on average during a 24-hour period, and experiencing serious psychological distress were more prevalent among adults with IBD than among those without IBD, as were several chronic conditions, including cardiovascular disease, respiratory disease, cancer, arthritis, weak or failing kidneys, any liver condition, and ulcer.

**What are the implications for public health practice?**

Adults with IBD who have mild to moderate disease activity should be encouraged to consult their clinicians about their exercise engagement. Clinicians should be aware of potential adverse health consequences of the health-risk behaviors that are more prevalent among adults with IBD, such as having insufficient sleep. Because certain chronic conditions are more prevalent among adults with IBD, disease management might involve multidisciplinary clinical care.

## Footnotes

* https://www.cdc.gov/nchs/nhis/index.htm.

† ftp://ftp.cdc.gov/pub/Health_Statistics/NCHS/Dataset_Documentation/NHIS/2015/srvydesc.pdf; ftp://ftp.cdc.gov/pub/Health_Statistics/NCHS/Dataset_Documentation/NHIS/2016/srvydesc.pdf.

§ Cigarette smoking status was defined as current, former, or never smoker. Current smokers reported having smoked ≥100 cigarettes in their lifetime and currently smoking cigarettes some days or every day. Former smokers reported having smoked ≥100 cigarettes in their lifetime but were not current smokers at the time of the survey. Never smokers reported they had not smoked ≥100 cigarettes in their lifetime. Binge drinking was defined as ≥12 heavy drinking days (five or more alcoholic drinks for men and four or more alcoholic drinks for women) in the past year. BMI (kg/m^2^) was categorized as underweight (<18.5), normal weight (≥18.5 and <25.0), overweight (≥25.0 and <30.0), or obese (≥30.0). The definition of physical activity categories followed the *2008 Physical Activity Guidelines for Americans* (https://health.gov/paguidelines/pdf/paguide.pdf). Both aerobic and muscle-strengthening guidelines are met if participants reported ≥150 minutes of moderate or ≥75 minutes of vigorous equivalent aerobic activity per week and muscle strengthening activities on ≥2 days per week. Short sleep duration was defined as reporting <7 hours of sleep, on average, in a 24-hour period.

¶ Serious psychological distress is based on responses to six questions that ask how often a respondent experienced certain symptoms (feeling so sad nothing could cheer you up; nervous; restless or fidgety; hopeless; that everything was an effort; or worthless) of psychological distress during the past 30 days. The response codes (0–4) of the six items for each person are summed to yield a scale with a 0–24 range. A value of ≥13 for this scale is used here to define serious psychological distress.

** Cardiovascular disease included a history of any of the following conditions: coronary heart disease, angina, myocardial infarction, stroke, or any heart disease. Respiratory disease included a history of any of the following conditions: emphysema, chronic bronchitis, chronic obstructive pulmonary disease, or asthma. Cancer included cancer or a malignancy of any kind. Diabetes was defined as an affirmative response to the question “Other than during pregnancy, have you ever been told by a doctor or other health professional that you have diabetes or sugar diabetes?” Arthritis was defined as an affirmative response to the question “Have you ever been told by a doctor or other health professional that you have some form of arthritis, rheumatoid arthritis, gout, lupus, or fibromyalgia?” Weak or failing kidneys was defined as an affirmative response to the question “During the past 12 months, have you been told by a doctor or other health professional that you had weak or failing kidneys? Do not include kidney stones, bladder infections or incontinence.” Any liver condition was defined as an affirmative response to the question “During the past 12 months, have you been told by a doctor or other health professional that you had any kind of liver condition?” Ulcer was defined as an affirmative response to the question “Have you ever been told by a doctor or other health professional that you had an ulcer?”

†† The relative standard error is equal to the standard error divided by the estimate, then multiplied by 100.

§§ Age-adjusted prevalence analysis used the projected 2000 U.S. population distribution #8 (18–24 years, 25–44 years, 45–64 years, and ≥65 years). https://www.cdc.gov/nchs/data/statnt/statnt20.pdf.

¶¶ http://www.crohnscolitisfoundation.org/assets/pdfs/ibdfactbook.pdf.

*** https://www.cdc.gov/sleep/data_statistics.html.
